# Human multi-organ chip co-culture of bronchial lung culture and liver spheroids for substance exposure studies

**DOI:** 10.1038/s41598-020-64219-6

**Published:** 2020-05-12

**Authors:** Katharina Schimek, Stefan Frentzel, Karsta Luettich, David Bovard, Isabel Rütschle, Laura Boden, Felix Rambo, Hendrik Erfurth, Eva-Maria Dehne, Annika Winter, Uwe Marx, Julia Hoeng

**Affiliations:** 1 TissUse GmbH, Oudenarder Str. 16, 13347 Berlin, Germany; 2 PMI R&D, Philip Morris Products S.A., Quai Jeanrenaud 5, 2000 Neuchâtel, Switzerland

**Keywords:** Respiratory system models, Lab-on-a-chip, Tissue engineering

## Abstract

Extrapolation of cell culture-based test results to *in vivo* effects is limited, as cell cultures fail to emulate organ complexity and multi-tissue crosstalk. Biology-inspired microphysiological systems provide preclinical insights into absorption, distribution, metabolism, excretion, and toxicity of substances *in vitro* by using human three-dimensional organotypic cultures. We co-cultured a human lung equivalent from the commercially available bronchial MucilAir culture and human liver spheroids from HepaRG cells to assess the potential toxicity of inhaled substances under conditions that permit organ crosstalk. We designed a new HUMIMIC Chip with optimized medium supply and oxygenation of the organ cultures and cultivated them on-chip for 14 days in separate culture compartments of a closed circulatory perfusion system, demonstrating the viability and homeostasis of the tissue cultures. A single-dose treatment of the hepatotoxic and carcinogenic aflatoxin B_1_ impaired functionality in bronchial MucilAir tissues in monoculture but showed a protective effect when the tissues were co-cultured with liver spheroids, indicating that crosstalk can be achieved in this new human lung–liver co-culture. The setup described here may be used to determine the effects of exposure to inhaled substances on a systemic level.

## Introduction

The often-limited translatability of data obtained in preclinical *in vivo* studies to the human situation and the restricted physiological relevance of current *in vitro* assays call for more predictive test methods. Microphysiological systems are *in vitro* models that better reflect the cellular microenvironment found *in vivo*. In comparison with conventional static *in vitro* organ models, medium flow in microphysiological systems removes microenvironments that can form around the cultures, improving nutrient and oxygen supply. Moreover, organ perfusion enables the creation of microenvironmental biomolecular gradients and relevant mechanical cues. These systems are regarded as ground-breaking in preclinical validation of substances and have the potential to change and accelerate drug development significantly. The number of publications on this topic has increased steadily since the first single-organ-on-a-chip study published by Michael Shuler’s group in 2004^1^. In addition to a wide variety of publications on single-organ-on-a-chip systems^2–4^, reports of multi-organ chips (MOCs) are increasing, as they can also emulate organ–organ crosstalk. Here, several organ equivalents are connected by microfluidic channels and can interact through the flow of culture medium. This, furthermore, allows investigation of pharmacokinetic parameters, such as absorption, distribution, metabolism, and excretion^5–8^. Successful homeostatic long-term organotypic co-cultures of human skin samples with three-dimensional (3D) human liver spheroids on a commercially available MOC platform have already been described^9^. Other MOC-based long-term co-cultures of various organ combinations have been established, such as liver spheroids with human 3D intestinal^10^, neuronal^11^, and pancreatic islet^12^ tissue models and a skin–intestine–liver–kidney^13^ chip for complex multi-tissue testing of substances. We recently reported a first repeated-dose test for simultaneous generation of safety and efficacy data using a MOC platform adapted for co-culturing human H292 lung cancer microtissues and human full-thickness skin equivalents^14^.

Here, we describe a novel MOC connecting a 3D air–liquid interface bronchial model with liver spheroids to assess the potential toxicity of inhaled substances under conditions that permit organ crosstalk. Such 3D bronchial models can be generated by cultivating human primary bronchial epithelial cells on porous carriers at the air–liquid interface^15^. Once exposed to air, the cells will differentiate and pseudostratify, forming a 3D tissue resembling the *in vivo* tissue. These models have been widely used to mimic human respiratory diseases, such as chronic obstructive pulmonary disease, asthma, rhinosinusitis, and cystic fibrosis^16^. Because of the latest developments in microphysiological system engineering, it is now also possible to imitate blood circulation and physical movements of the tissues, which, for example, influence the permeability of bronchial epithelium^17–19^. In the context of toxicological assessment, these 3D lung models cultured under conventional static conditions are inaccurate, as they only replicate the primary effects of inhaled substances, and the toxicity of these substances can be influenced by other organs once in the bloodstream.

The liver expresses approximately 30 cytochrome P450 (CYP) enzymes and, therefore, plays a decisive role in metabolizing substances^20^. Several groups have developed platforms to mimic the human liver in combination with other organs such as the lungs^1,21,22^. Major efforts in this area have been directed towards drug metabolism and toxicity studies by using primary human liver cells or hepatoma cell lines^23,24^. In this study, we used spheroids formed from HepaRG cells and primary human hepatic stellate cells (HHSteCs). HepaRG is a carcinoma cell line that bears characteristics similar to those of primary human hepatocytes and has been used successfully in drug metabolism and toxicity studies^25–27^. These cells have also been shown to form 3D spheroids with better functional performance than that of standard two-dimensional models in static cultures^27–29^.

In the present study, we demonstrated the stability and functionality of the two models cultured in a specifically designed MOC over 14 days. This MOC included a large medium reservoir and an air-permeable membrane above the lung culture compartment to ensure optimal air circulation. The surface area of the bronchial MucilAir model used was 0.33 cm^2^, corresponding to 1/100,000 of the surface area of the entire respiratory tree without the alveolar surface area. The number of liver cells used per chip represents a similar proportion in a normal human liver. Aflatoxin B_1_ (AFB_1_) was used to demonstrate organ interaction. We found that the toxicity of 5 µM AFB_1_ decreased in the presence of the liver spheroids, confirming that this lung–liver co-culture system can be used to evaluate the toxicity of inhaled substances.

## Results

### Oxygen pressure (pO_2_) dynamics in the Chip3plus

**Figure 1:**
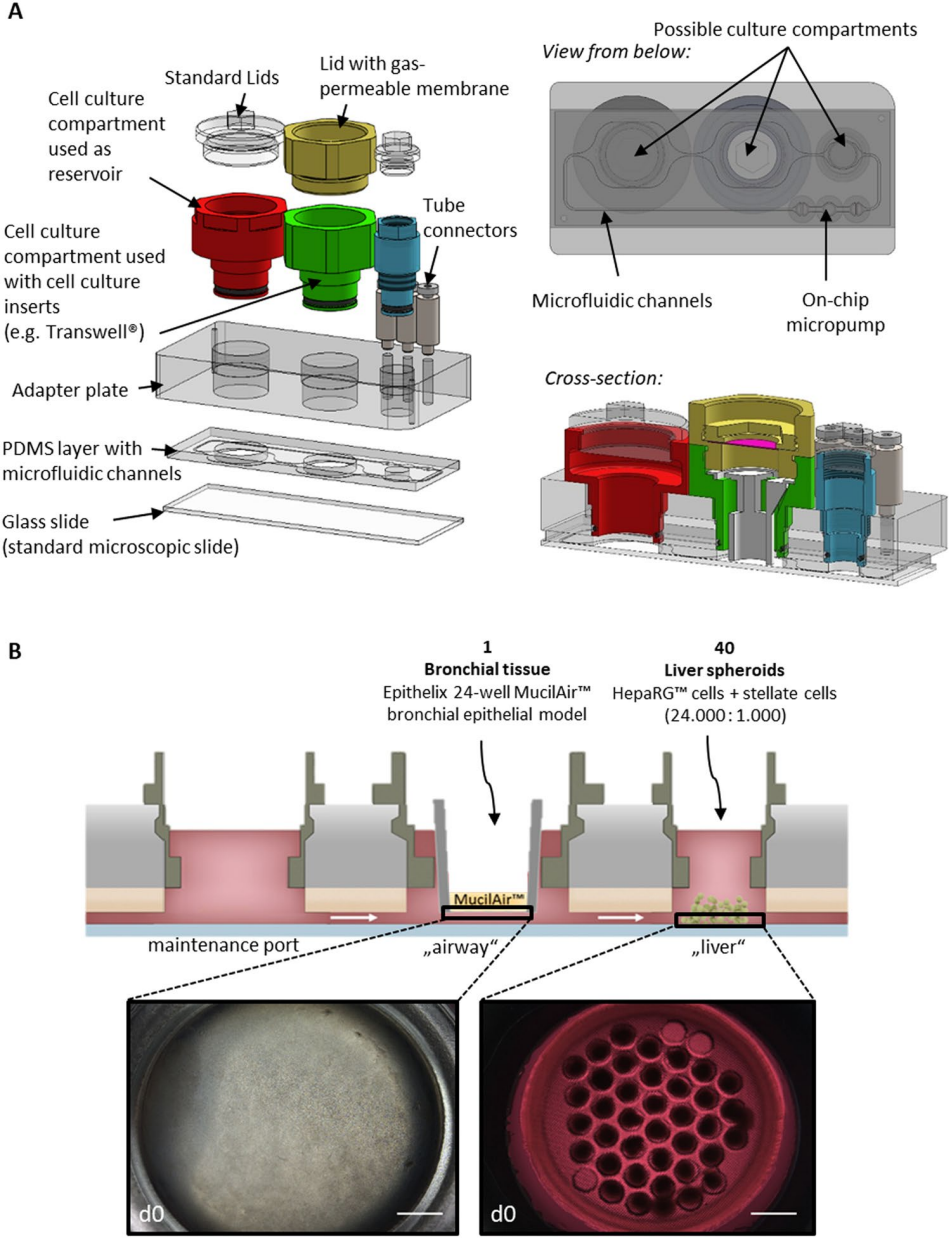
The multi-organ chip platform and experimental setup. (**A**) Components of the Chip3plus optimized for medium supply, illustrated by exploded, bottom, and cross-sectional views of the chip. (**B**) Relevant organ equivalents and loading scheme for Chip3plus culture. Forty liver spheroids and one bronchial MucilAir culture were placed in one Chip3plus circuit (sectional representation). Clockwise, pulsatile pumping direction; the medium passes through the medium reservoir and then through the MucilAir culture (middle) and liver model. Brightfield images show the bronchial MucilAir culture (left) and liver spheroids embedded into a SpheroidBrick inlay in their respective culture compartments on day 0 (d0). Scale bar: 1000 µm.

**Figure 2:**
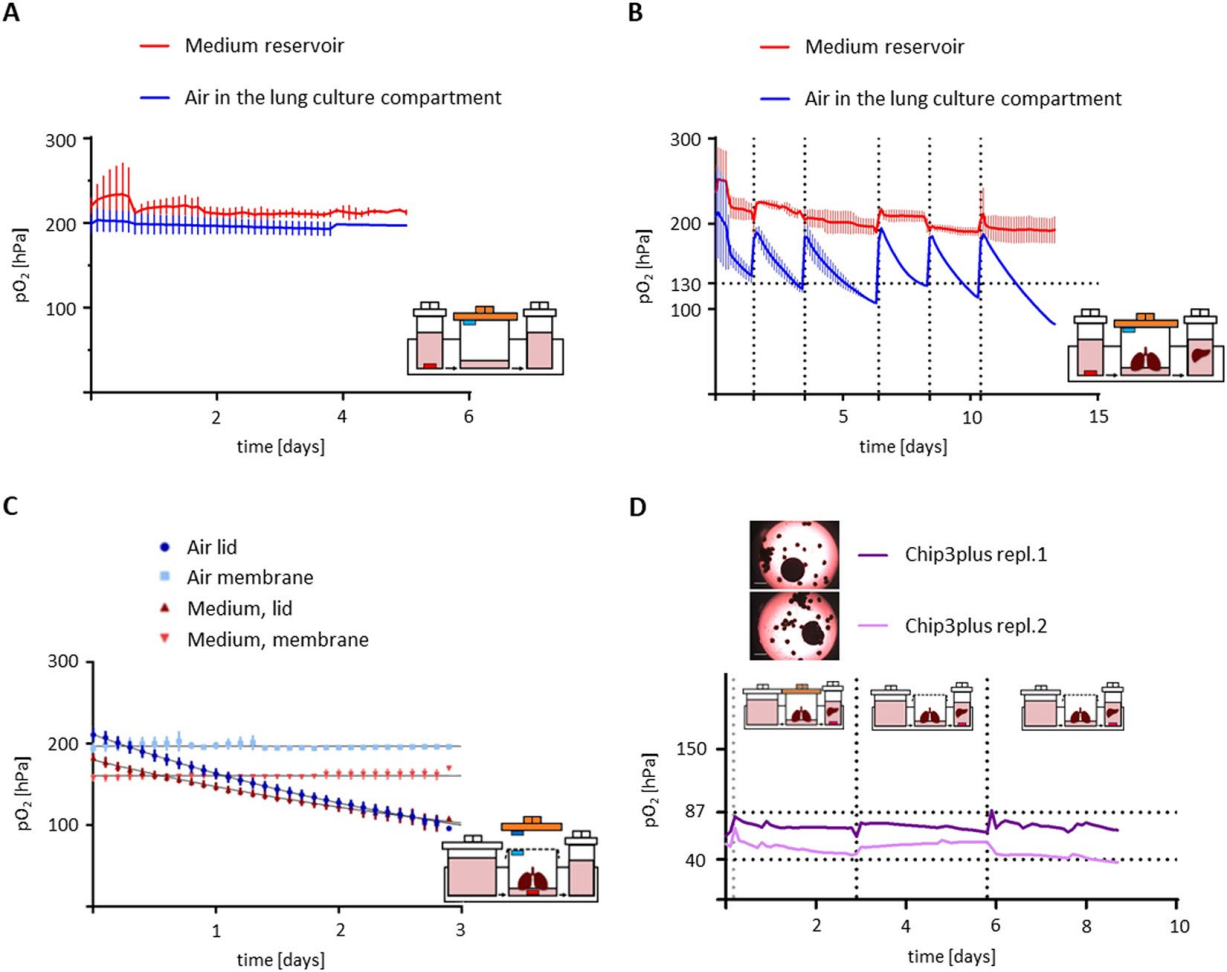
Online oxygen measurement in the Chip3plus. (**A,B**) Oxygen pressure (pO_2_) dynamics in the reservoir medium (red line) and lung compartment air (blue line) (**A**) in a cell-free Chip3plus and (**B**) during lung–liver co-culture, shown as mean ± standard deviation (N = 2). The pO_2_ was stable in the cell-free chip over at least 5 days but fell below 130 hPa (dotted horizontal line) in the lung compartment during co-culture 2 days after each medium exchange (dotted vertical lines). The pO_2_ remained stable at atmospheric pO_2_ levels in the medium reservoir. (**C**) pO_2_ dynamics in the air and medium of the lung compartment during lung monoculture using airtight lids or gas-permeable membranes as covers, shown as mean ± standard deviation (N = 3–4). (**D**) pO_2_ dynamics in the liver compartment in a lung–liver co-culture. Days 0–3: The lung compartment was covered with an airtight lid. Days 3–9: The lung compartment was covered with a gas-permeable membrane. Black dotted vertical lines indicate medium exchanges, and the grey dotted vertical line indicates the commencement of pumping. Dotted horizontal lines indicate physiological pO_2_ values for blood entering the periportal zone of a human liver sinusoid (80–87 hPa) and leaving the pericentral zone (40–47 hPa).

A HUMIMIC Chip3plus (TissUse GmbH, Berlin, Germany) was designed which allows for the co-culture of lung and liver tissues to obtain more complex and physiologically relevant tissue responses after exposure to inhaled toxicants (Fig. 1). Tissue culture conditions such as pH, temperature, and oxygen supply affect cell behaviour and, therefore, emulation of the *in vivo* situation. We, therefore, monitored the pO_2_ and its dynamics using oxygen sensors (PreSens Precision Sensing GmbH, Regensburg, Germany) at various locations inside the Chip3plus. The pO_2_ was measured at the bottom of the medium reservoir to determine partial pO_2_ within the medium and underneath the lid of the lung culture compartment to monitor pO_2_ in the air inside the chip (Fig. 2A). First, baseline levels in a cell-free Chip3plus were investigated over 5 days, and the pO_2_ remained stable at both locations over the cultivation period. The pO_2_ was measured as 200 hPa in the air under the lid and 210–220 hPa in the culture medium, indicating that the atmospheric pO_2_ value outside the incubator (approximately 215 hPa, 21% oxygen at an average pressure of about 1 atm) was maintained in the Chip3plus over the testing period. We noted that the pO_2_ values did not decrease to match the pO_2_ value in the incubator (185–192 hPa, depending on the air pressure). It can, therefore, be assumed that gas diffusion from the incubator air through the polydimethylsiloxane (PDMS) channel walls of the chip did not occur. This indicates the presence of oxidized PDMS, which was reported to have a significant smaller diffusion coefficient of the oxygen compared to non-oxidized PDMS^30^.

The experiment was repeated over a period of 14 days with the lung–liver co-culture (Fig. 2B). The cell culture medium was exchanged three times a week to ensure sufficient nutrient supply to the tissues. This required opening of the culture compartments under sterile conditions. Consequently, the air inside the chip was replaced with fresh room air at each medium renewal, and the pO_2_ returned to atmospheric levels. The air pO_2_ dropped rapidly in the lung culture compartment between medium exchanges. Within 2 days, we observed a decrease from 200 hPa to below 130 hPa, which is the lowest pO_2_ detected in the lungs *in vivo* at sea level and occurs in the alveoli^31^. The pO_2_ in the medium reservoir remained relatively constant between medium exchanges, at around 200 hPa.

Given the rapid decrease in pO_2_ observed in the air of the lung culture compartment (Fig. 2B), we investigated the effect of a gas-permeable membrane covering the compartment instead of using an airtight lid on oxygenation (pO_2_). In the first setting, the pO_2_ was measured only in the presence of the bronchial MucilAir culture, followed by an experiment where both tissue models—a bronchial MucilAir culture and 40 liver spheroids—were in the chip. Again, the pO_2_ in the medium was measured at the bottom of the medium reservoir and underneath the lid/membrane of the lung culture compartment. The results for the air pO_2_ underneath the lid obtained previously were reproduced in the lung monoculture with lid (Fig. 2C). In contrast to the airtight lid, the gas-permeable membrane maintained the air pO_2_ inside the lung culture compartment stable at about 200 hPa. In addition to the pO_2_ drop in the lung culture compartment air, the airtight lid also caused a decrease in the pO_2_ in the medium below the bronchial MucilAir culture. The use of a membrane stabilized the pO_2_ in the culture medium to a value of 160 hPa, which is below the pO_2_ values described above for the air and in the medium reservoir. This indicates that oxygen consumption by the bronchial MucilAir culture exceeded the reoxygenation achievable by medium circulation and O_2_ diffusion.

Subsequently, we investigated the pO_2_ dynamics in the liver culture compartment. Two replicates of a lung–liver co-culture were used. These were initially cultivated for 3 days with tight plastic lids on the lung culture compartment and then with membranes for 6 days. Over time, the pO_2_ in the culture medium remained stable on each chip at a comparatively low level of 70–87 hPa for replicate 1 and 40–50 hPa for replicate 2 (Fig. 2D). After starting the pump, the pO_2_ value in both chips increased by 30%. In contrast, the variability between the chips in the liver culture compartment was quite large. Furthermore, the difference in pO_2_ observed between the medium reservoir (Fig. 2B) and liver compartment (Fig. 2D) confirmed that the cells in the liver compartment were metabolically active and consumed oxygen. From these measurements, it can also be assumed that spatial pO_2_ gradients were present in the medium circulating in the chip. No differences in pO_2_ were observed between liver culture compartments under the airtight lid and the gas-permeable membrane.

### Evaluation of liver monocultures in the Chip3plus

**Figure 3:**
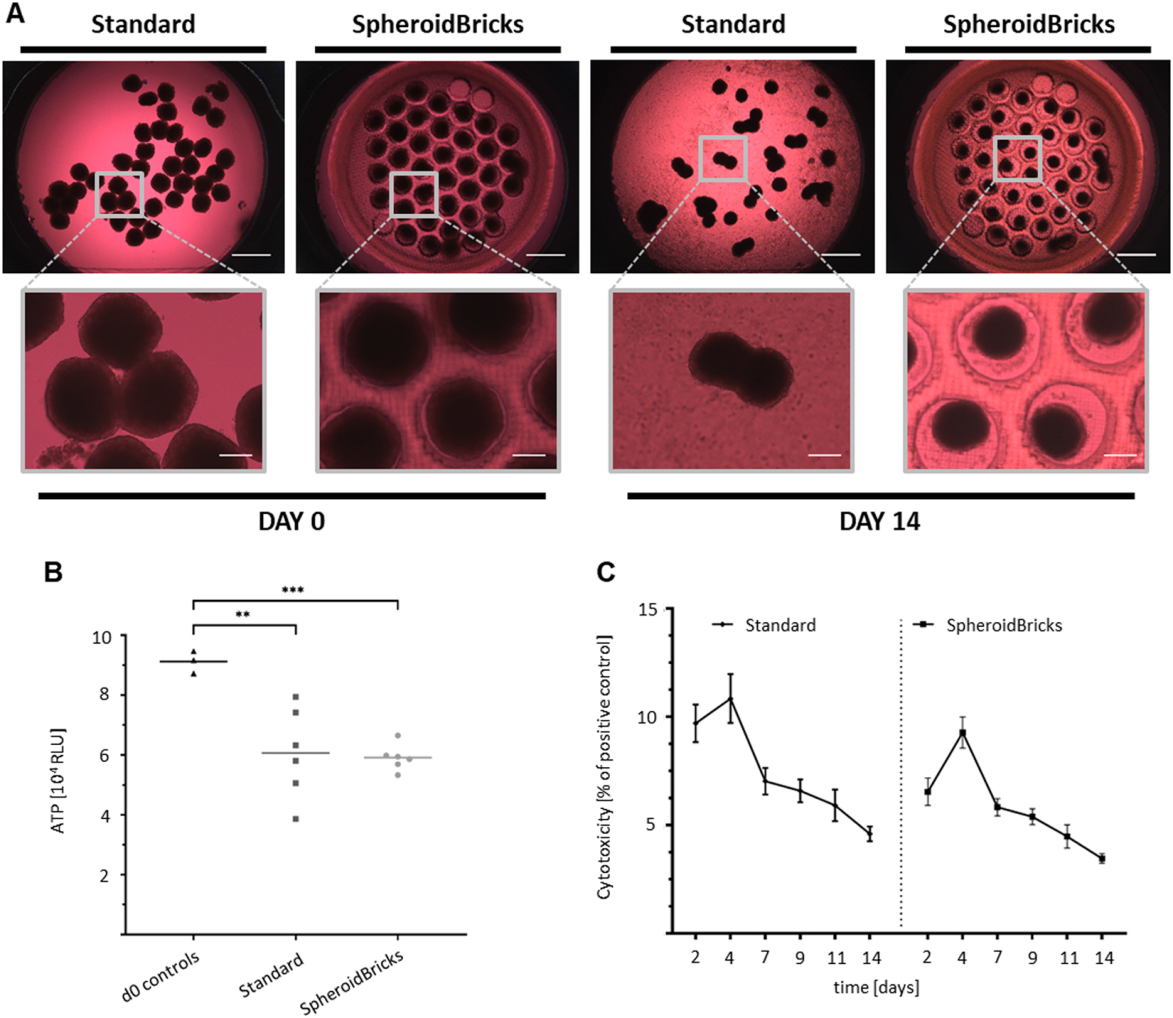
Comparison of liver spheroid cultures in the Chip3plus. (**A**) Representative images of the liver culture compartment illustrating a typical arrangement of liver spheroids on days 0 and 14, cultured under either standard conditions (on the glass surface) or in SpheroidBrick inlays. Under standard culture conditions without SpheroidBrick inlays, the liver spheroids fused, and the cells spread on the glass surface of the Chip3plus. Scale bars: 1000 µm (overview) and 250 µm (magnified images). (**B**) ATP-based viability assay performed with single liver spheroids extracted from Chip3plus cultures of standard and SpheroidBrick culture conditions and compared with viability in day 0 controls. Liver spheroids cultured in the SpheroidBrick inlays were more homogeneous in total adenosine triphosphate content. **p < 0.01, ***p < 0.001 by using the Brown–Forsythe and Welch ANOVA tests with Dunnett’s T3 multiple-comparison post-hoc test. (**C**) Lactate dehydrogenase (LDH) release from cultured tissues over 14 days shown in relation to the total LDH level of a positive control (Triton X-100 treated tissues). The Chip3plus standard culture is compared with a Chip3plus culture with SpheroidBrick inlays; the cultures displayed a similar cytotoxicity profile. Data shown as mean ± standard deviation (N = 3–8).

We then evaluated various culture conditions using liver spheroids in the Chip3plus (Fig. 3A). When cultivated on the glass surface of the Chip3plus (standard condition), the liver spheroids adhered after a few days, visible as a spread of cells across the glass surface. In some cases, the free-floating liver spheroids also fused, forming large tissues and possibly leading to the formation of necrotic cores. For this reason, we developed a novel approach to maintain the liver spheroids in the Chip3plus: A disk made from polyethylene glycol and containing 40 cavities, each with a diameter of 500 µm, was used as an inlay in the liver culture compartment of the Chip3plus; this was termed SpheroidBrick (Cellbricks GmbH, Berlin, Germany). This inlay is large enough for liver spheroids, while keeping each one separated by the cavity walls. The polyethylene glycol substrate also prevents the liver spheroids from adhering to the surface. As shown by adenosine triphosphate (ATP) measurement, the liver spheroids cultured in the SpheroidBricks were more homogeneous at harvesting than those cultured by using conventional culture methods (Fig. 3B). Lactate dehydrogenase (LDH) release, used as a marker of cytotoxicity, confirmed that the polyethylene glycol-based SpheroidBricks had no major effect on cytotoxicity, as it remained below 10% and even decreased under both conditions over 14 days (Fig. 3C).

### Long-term stability of lung–liver co-cultures

**Figure 4:**
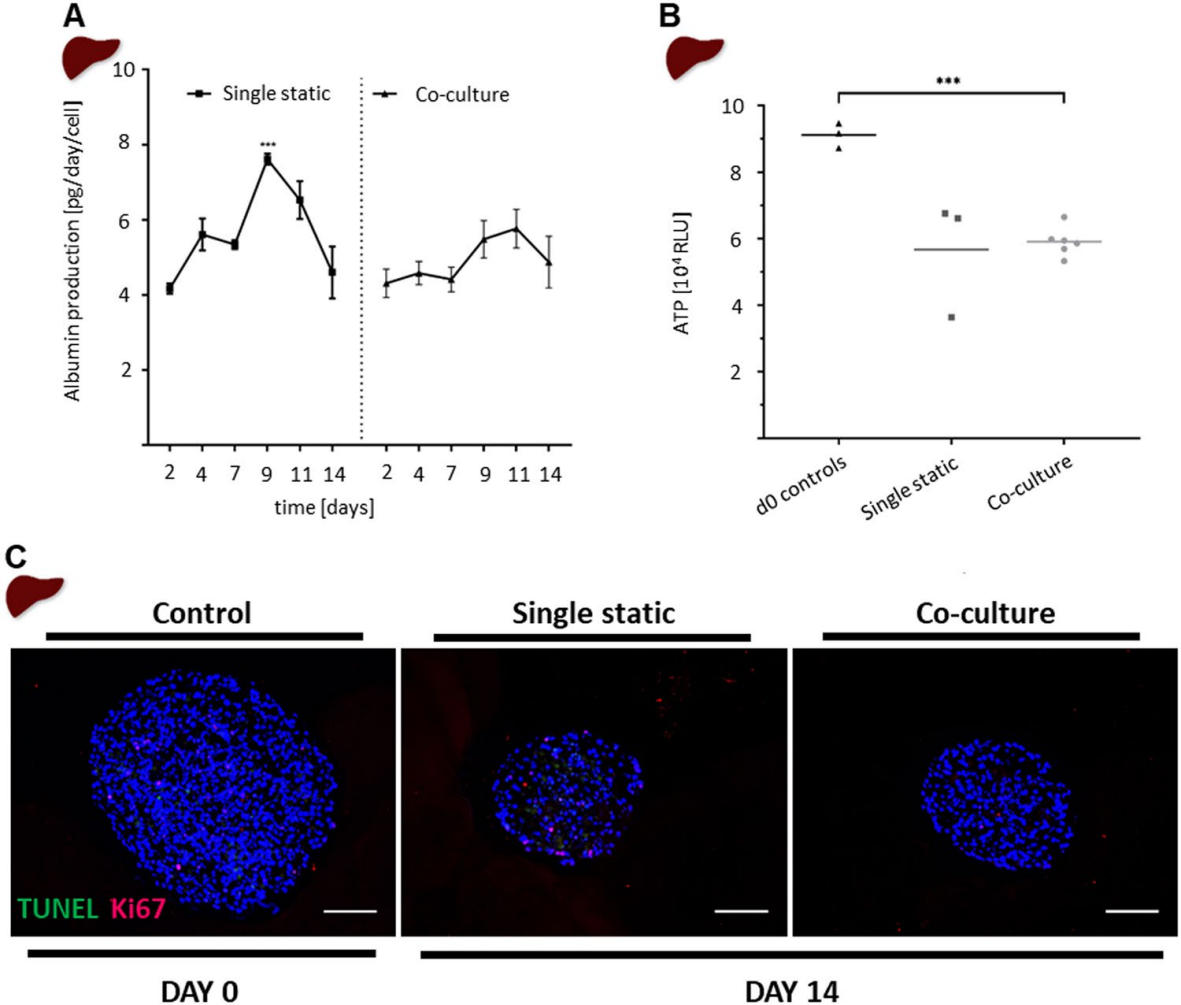
Comparison of static and co-cultured liver spheroids. (**A**) Albumin production was measured every 48–72 h in medium collected during 50% medium exchange. Co-cultures (right; triangle) are compared with monocultures (left; square). ***p < 0.001 by using mixed-effects analysis with Tukey’s multiple-comparison post-hoc test. (**B**) The adenosine triphosphate-based viability assay performed with single liver spheroids extracted from static culture or Chip3plus co-culture and compared with viability in day 0 controls. ***p < 0.001 using the Brown-Forsythe and Welch’s ANOVA tests with Dunnett’s T3 multiple-comparison post hoc test. (**C**) tdT-mediated dUTP-digoxigenin nick-end labelling (green, apoptotic cells) and Ki67 (red, proliferative cells) double-staining of liver spheroids. Scale bar: 100 µm. Data shown as mean ± standard deviation (N = 3–8).

Following the initial characterization and adaptation, the suitability of the chip for maintaining mono- or co-cultures for 14 days was assessed. Functional markers and the viability of the liver spheroids were assessed by albumin production, ATP content, and tdT-mediated dUTP-digoxigenin nick-end labelling (TUNEL)/Ki67 staining. The Chip3plus co-cultures were compared with statically cultured liver spheroids in monoculture and baseline (day 0) controls. The liver spheroids were cultured in SpheroidBricks to maintain their 3D morphology, and the HHSteCs were omitted from the spheroids, as HHSteC activation was assumed from upregulation of HHSteC markers (e.g., collagen I) after SpheroidBrick culturing (data not shown). Albumin production was relatively stable over the 14-day test period, at about 6 ± 2 pg/day per HepaRG cell, regardless of whether the cultures were maintained under static or dynamic conditions (Fig. 4A). Lung–liver co-cultures showed lower variability in albumin production over time than static liver monocultures. Moreover, the ATP levels in co-cultured liver spheroids were not substantially different from those in the statically maintained liver spheroid monocultures (Fig. 4B); however, a 35% decrease from baseline was observed under both conditions. These findings support the morphological (data not shown) and immunohistochemical (Fig. 4C) observations that the spheroids cultured under both conditions appeared to become smaller and less dense during culturing. However, liver spheroids from both conditions showed comparable numbers of proliferating cells positively stained for Ki67 and low numbers of apoptotic cells positively stained for TUNEL.

**Figure 5:**
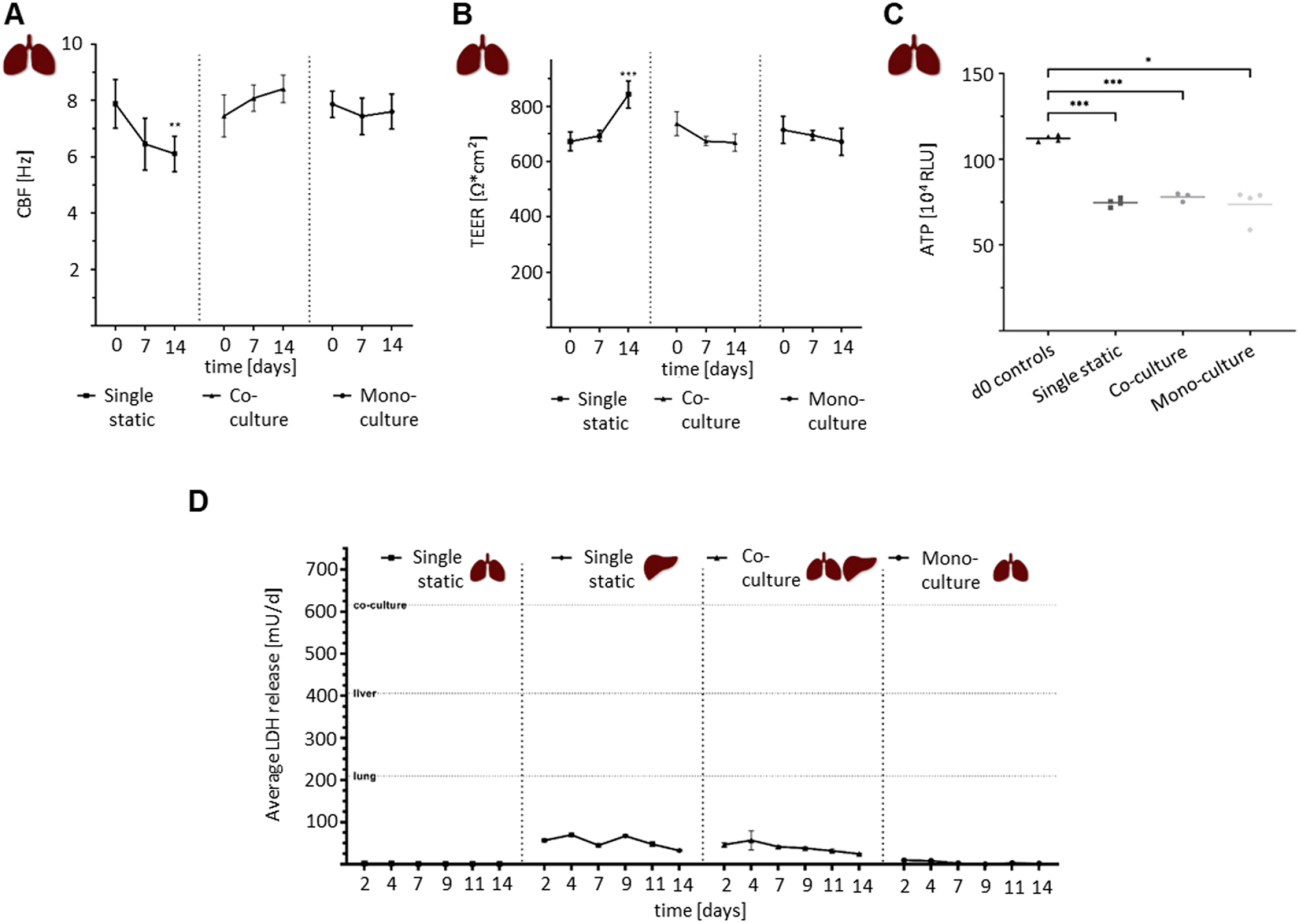
Further evaluation of Chip3plus co-culture experiment. (**A–C**) Comparison of static lung monoculture, Chip3plus lung–liver co-culture, and Chip3plus lung monoculture. (**A**) Ciliary beating frequency (CBF) and (**B**) transepithelial electrical resistance (TEER) measurements on days 0, 7, and 14 of culture. **p < 0.01, ***p < 0.001 by using mixed-effects analysis with Tukey’s multiple-comparison post-hoc test. (**C**) The adenosine triphosphate-based viability assay performed with bronchial MucilAir cultures cultivated for 14 days and compared with viability in day 0 controls. *p < 0.05, **p < 0.01, ***p < 0.001 by using the Brown–Forsythe and Welch ANOVA tests with Dunnett’s T3 multiple-comparison post-hoc test. (**D**) Lactate dehydrogenase (LDH) release in all culture conditions over 14 days. Static lung and liver monocultures are compared with Chip3plus lung–liver co-cultures and Chip3plus lung monocultures. Horizontal lines indicating positive controls (maximum LDH release values) of the respective condition. Data shown as mean ± standard deviation (N = 3–8).

Bronchial MucilAir cultures evaluated through ATP measurement showed that the culturing method (static liver monoculture vs. Chip3plus lung–liver co-culture vs. Chip3plus lung monoculture) and 14-day culturing period had no impact on tissue viability (Fig. 5C). The functionality of bronchial MucilAir cultures was assessed by measuring the ciliary beating frequency and transepithelial electrical resistance. Ciliary beating is a key mechanism of mucociliary clearance and airway defence against inhaled particles and pathogens^32^. Transepithelial electrical resistance is a marker of tight junctions and biological barrier integrity^33^. Bronchial MucilAir tissues cultured alone in the Chip3plus were compared with tissues co-cultured with liver spheroids and those cultured under static conditions. The ciliary beating frequency of bronchial MucilAir tissues in static monoculture decreased by approximately 20% from baseline (Fig. 5A), while the transepithelial electrical resistance increased by about 20% at the same time (Fig. 5B). The tissues maintained in the chip under medium flow exhibited no significant changes in transepithelial electrical resistance or ciliary beating frequency over the 14-day culturing, regardless of whether they had been cultured alone or in co-culture.

LDH release into the culture medium was assessed for all four conditions (static lung and liver monocultures, Chip3plus lung–liver co-culture, and Chip3plus lung monoculture). LDH release is widely used as a measure of cell death, as this soluble cytoplasmic enzyme is present in almost all cell types and released into the extracellular space when the plasma membrane is damaged^34^. A release of about 50 mU LDH/day was detected on the second day of co-culturing, and decreased continuously from day 4 to a 50% lower LDH release on day 14. The same was observed in the liver monocultures, whereas lung monocultures, regardless of their culturing method (static vs. on-chip), exhibited almost no release of LDH into the basolateral medium. It can, therefore, be assumed that the LDH measured in the co-cultures originated primarily from the liver spheroids.

These findings demonstrated that both models could be maintained in co-culture over the 14-day period and that specific organ functions remain relatively stable.

### Assessment of AFB1 toxicity in lung–liver co-cultures

**Figure 6:**
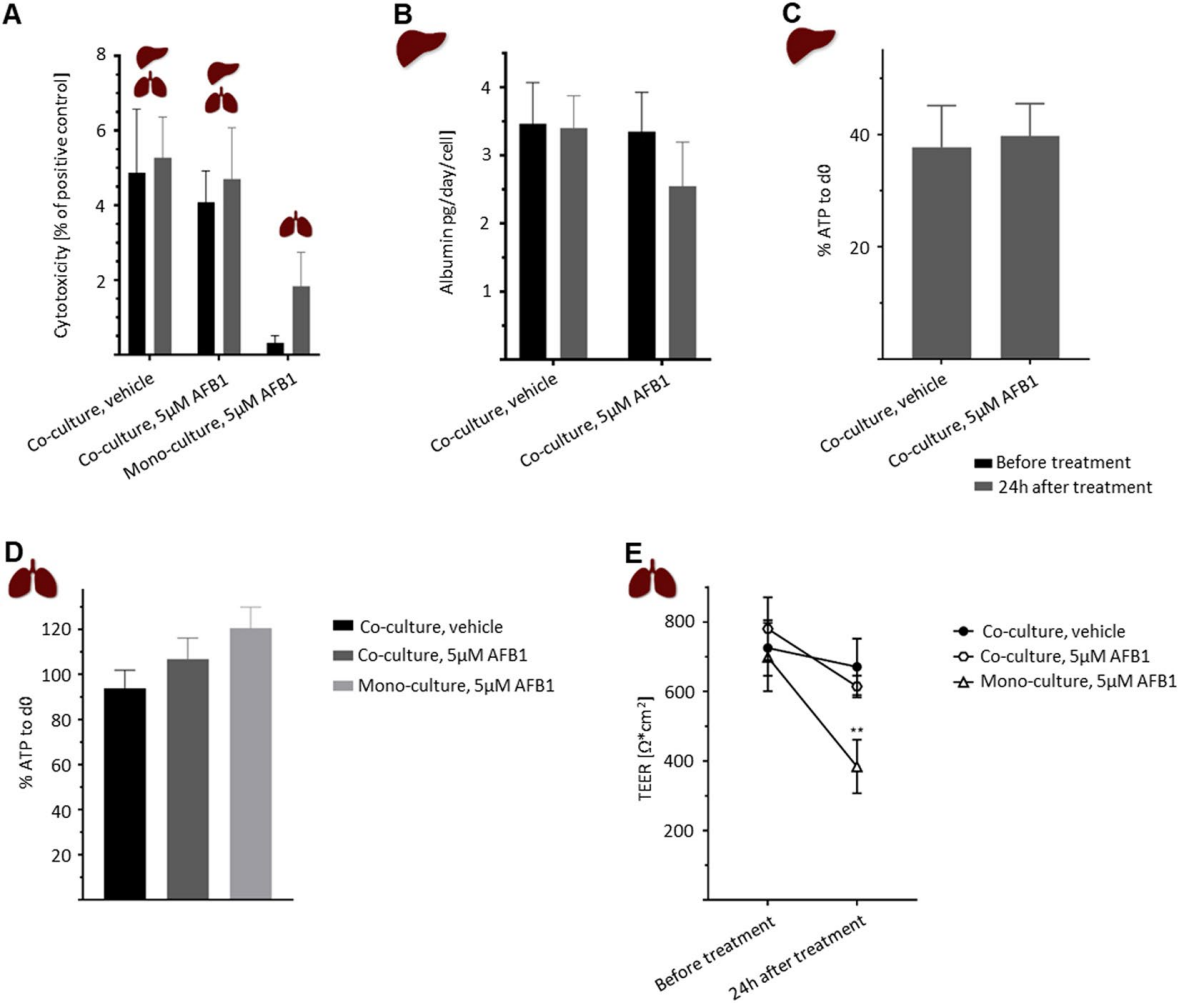
Effects of treatment with 5 µM aflatoxin B_1_ (AFB_1_) on bronchial MucilAir cultures and liver spheroids in Chip3plus lung–liver co-culture or lung monoculture. (**A**) Cytotoxicity (measured by lactate dehydrogenase release) in co-cultures treated with vehicle control, co-cultures treated with AFB_1_, and lung monoculture treated with AFB_1_ before (black bars) and 24 h after (grey bars) treatment. (**B**) Albumin production in co-cultures before (black bars) and 24 h after (grey bars) treatment with vehicle or AFB_1_. (**C**) Adenosine triphosphate (ATP) content of co-cultured liver spheroids treated with vehicle or AFB_1_, normalized to the ATP content in day 0 controls. (**D**) ATP content of co-cultured bronchial MucilAir cultures treated with vehicle or AFB_1_ and monocultured bronchial MucilAir cultures treated with AFB_1_, normalized to the ATP content in day 0 controls. (**E**) Transepithelial electrical resistance measured in co-cultured bronchial MucilAir cultures treated with vehicle or AFB_1_ and monocultured bronchial MucilAir cultures treated with AFB_1_ before and 24 h after treatment. **p < 0.01 by using mixed-effects analysis with Tukey’s multiple-comparison post-hoc test. Data shown as mean ± standard deviation (N = 3–4).

AFB_1_ was selected to demonstrate the crosstalk of the lung–liver co-culture in Chip3plus and its suitability for substance testing. AFB_1_ is a hepatotoxic compound produced by *Aspergillus* moulds. Its cytotoxicity and subsequent detoxification are mediated via metabolic activation by drug-metabolizing CYP enzymes^24,35–37^ and has also been shown to occur in human airways^38^. Moreover, this compound was used recently in a lung–liver chip described by Bovard *et al*., where cytotoxicity in normal human bronchial epithelial cells cultured at the air–liquid interface was delayed by co-culture with liver spheroids^21^. We exposed the lung–liver co-cultures and lung monocultures to 5 µM AFB_1_ after two days of culture in the chip to allow the tissues to adapt to the flow conditions and investigate the short-term effects after 24 h of exposure. This concentration was selected because it is in the range of the half-maximal threshold concentration described for AFB_1_ treatments of HepaRG cultures^24,39^. After 24 h of exposure to 5 µM AFB_1_, a decrease of 1 pg/day/cell in albumin production was observed in the lung–liver co-cultures, indicating impairment of liver function (Fig. 6B). In contrast, intracellular ATP levels in co-cultured liver spheroids treated with AFB_1_ (Fig. 6C) and LDH release into the co-culture medium (Fig. 6A) were unchanged from those in vehicle controls. Co-cultured bronchial MucilAir cultures treated with AFB_1_ showed a slight decrease in transepithelial electrical resistance (Fig. 6E) and a moderate increase in ATP content (Fig. 6D) compared with those observed in vehicle controls. Monocultured bronchial MucilAir cultures treated with 5 µM AFB_1_ exhibited a much stronger decline in transepithelial electrical resistance (Fig. 6E) than pre-treatment cultures and vehicle controls, and a higher LDH release into the medium than cultures before treatment (Fig. 6A), indicating a greater decrease in functionality and viability than that observed in co-cultured bronchial MucilAir cultures. Interestingly, increased ATP content was observed in monocultured bronchial MucilAir cultures treated with AFB_1_, similar to that seen in the co-cultured models (Fig. 6D).

## Discussion

Inhaled substances do not only have an influence on the lungs. Their potential biological impact also depends on an interplay with the other organs, especially the liver, which is a key metabolizer, transforming compounds into metabolites with new properties. We present here a HUMIMIC Chip3plus that combines a physiologically relevant 3D lung model previously used for assessment of aerosol toxicity^40,41^ with liver spheroids acting as a metabolizing compartment.

Oxygen pressure is important for differentiation and maintenance of cellular models, as it has been shown to modulate differentiation of both ciliated cells and hepatocytes^42,43^. Hence, we characterized partial pO_2_ in the Chip3plus. In the lung compartment, we found that atmospheric pO_2_ decreased to 100 hPa within 3 days when the compartment was closed with an airtight lid. As this value is below physiological levels (130 hPa in the alveoli to 153 hPa in the upper airways at sea level)^31^, the Chip3plus lung compartment was modified. Using a gas-permeable membrane as a lid helped stabilize the pO_2_ close to physiological levels. Moreover, the pO_2_ in the liver compartment was in the range of 40–87 hPa. Blood entering the periportal zone of a human liver sinusoid contains oxygen with a pO_2_ of 80–87 hPa^44^; by the time the blood leaves the pericentral zone, the pO_2_ would have decreased to 40–47 hPa^44^. Thus, the pO_2_ values recorded in the culture medium around the liver spheroids were in the range of *in vivo* pO_2_. Because of the large variations in pO_2_ values measured in the liver compartment, the existence of special gradients within the compartment can be presumed. Therefore, measurements performed at one location can only be considered as an estimate of the pO_2_ for the whole compartment. Measurement variability may result from the non-homogeneous spreading of liver spheroids when they were cultured, in accordance with our standard protocol, at the bottom of the culture compartment. The number of liver spheroids between the microfluidic channel transporting medium from the direction of the lung compartment and the oxygen sensor may influence the pO_2_ measured at the centrally located sensor. Lower pO_2_ measurements, therefore, would indicate more oxygen-consuming liver spheroids located before the sensor. The variability in pO_2_ measurements could be improved in the future by using the SpheroidBrick inlays for culturing liver spheroids in chips. These have been shown here to result in a defined and more homogeneous distribution of liver spheroids in the liver compartment. Moreover, the SpheroidBricks prevented the adhesion of liver spheroids to the glass surface of the chip. The future use of SpheroidBricks for other spheroid-based tissue models is envisioned to yield higher reproducibility and reliability in multi-organ studies. Similarly, the use of oxygen sensors is applicable to other MOC-based cultures and will result in a better understanding of culture conditions and tissue behaviour. Additionally, the use of floating sensor beads in future studies may allow for spatially resolved oxygen measurements within the chip.

The present study yielded a robust microfluidic chip-based co-culture of human bronchial tissue and liver spheroids maintained for 14 days in a standardized cell culture medium. Chips have been described previously that enabled 14- to 28-day bronchial and liver tissue co-cultures^21,22^. Our chip differs in many respects including size, pump mechanism, and material. Although the use of PDMS is controversial because of its ability to absorb hydrophobic substances^45–47^, it renders our chip compatible with tissue imaging by its transparency and enables more rapid prototyping. Hence, the HUMIMIC Chip platform can be easily adapted to other organ combinations or to the addition of organs such as the kidney to replicate absorption, distribution, metabolism, excretion, and toxicity testing of inhaled compounds. Although the liver model used here is a robust proxy for human hepatocytes, replacing the HepaRG cells with primary human hepatocytes would improve the model and better reflect the functions of a human liver, enabling the selection of specific genotypes for studying inhaled substances^48^. Furthermore, the integration of other relevant cell types into the 3D models, such as Kupffer cells into the liver model or fibroblasts into the MucilAir culture, would provide a more accurate simulation of the pathophysiological response to a substance^49,50^.

The functional coupling of lung and liver models was evaluated by exposing the co-culture to AFB_1_. Decreased functionality was shown exclusively for the bronchial MucilAir culture in chip-based monocultures. It is widely accepted that the carcinogenic and acute toxic responses to AFB_1_ are dependent on its metabolic activation^35,36^. The major metabolites formed in the liver are AFB_1_–8,9-epoxide, aflatoxin M_1_–8,9-epoxide, and aflatoxin Q_1_^51^. Aflatoxin M_1_−8,9-epoxide and AFB_1_−8,9-epoxide have been shown to be cytotoxic, whereas aflatoxin Q_1_ is considered a detoxification product^52–54^. It is assumed that bioactivation in bronchial cells results exclusively in the production of cytotoxic metabolites^38,55,56^. We cannot conclude with absolute certainty that the AFB_1_ was metabolized because we did not assess the presence of metabolites in the chip microfluidics, and proof of activation-based AFB_1_ toxicity would require inhibition of all CYP enzymes in the tissues. Nonetheless, a protective decrease in AFB_1_ toxicity in the bronchial MucilAir culture by the metabolic capacity of the liver spheroids was shown following treatment with 5 µM AFB_1_. This finding demonstrates crosstalk in the new human lung–liver MOC system and reproduces the findings reported by Bovard *et al*.^21^, although the concentration of AFB_1_ for which an effect could be demonstrated was lower (5 µM vs. 100 µM). However, comparison of the data is difficult because of the different test conditions (e.g., composition and volume of cell culture medium). The use of other test substances will assist in evaluating the performance of the individual MOC platforms. In this regard, exposure of the lung tissue to an aerosol would be preferable for testing the MOC platform under a realistic exposure scenario. This will enable assessment of not only the toxicity but also the bioavailability of inhaled substances and could, therefore, become an important tool in drug discovery and personalized medicine.

## Materials and Methods

### Chip design and fabrication

A new design of the commercially available microphysiological HUMIMIC Chip platform^14^, Chip3plus, was developed to contain three consecutive culture compartments—one of a 96-well format and two of a 24-well format—thereby allowing an increase in the medium volume to up to 4 mL per circuit (Fig. 1A). In this study, the Chip3plus was used to co-culture one 24-well hanging Transwell insert-based bronchial MucilAir culture with 3D HepaRG-based liver spheroids (Fig. 1B). The device was fabricated as described by Wagner *et al*.^9^. Briefly, a PDMS layer of 2-mm height, containing the microfluidic channel, culture compartment openings, and micropump membranes, was cast against a polycarbonate adapter plate by replica moulding. The PDMS layer was then bonded to a microscopic glass slide by using low-pressure oxygen plasma, closing the channels at a height of 100 µm. A commercially produced batch of the Chip3plus was used for the experiments after passing quality control. Pulsatile fluid flow in the tissue-connecting circuit is driven by an on-chip micropump, modified after Wu and colleagues^57^. An external control unit (HUMIMIC Starter, TissUse GmbH) maintains a pulsatile flow in each circuit of the Chip3plus through alternating application of compressed air and vacuum to the pump membranes at a defined frequency. Each control unit can operate eight Chip3plus simultaneously. The on-chip peristaltic micropump was operated at a pumping frequency of 0.5 Hz and pressure of 50 kPa, enabling continuous pulsatile flow. At this frequency, the medium flow rate corresponded to 5.32 ± 1.46 µL/min. Microscopic inspection from the bottom of the Chip3plus permitted daily monitoring of tissue integrity.

### Cell sources and maintenance

Differentiated HepaRG cells (lot HPR116259-TA08 or HPR116NS080003) were obtained from Biopredic International (Rennes, France). The HHSteCs were purchased from ScienCell (Carlsbad, CA, USA). Bronchial MucilAir cultures were purchased from Epithelix Sàrl and consisted of tissues from a non-smoking healthy man (41-year-old Caucasian; batch MD072001) or a non-smoking woman (59-year-old Caucasian; batch MD076801). The differentiated HepaRG cells were thawed and seeded 7 days before spheroid formation. Standard HepaRG culture medium consisted of Williams’ E medium (PAN-Biotech, Aidenbach, Germany) supplemented with 10% foetal calf serum (Corning Life Sciences, Tewksbury, MA, USA), 5 μg/mL human insulin (PAN-Biotech), 2 mM L-glutamine (Corning), 5 × 10^−5^ M hydrocortisone hemisuccinate (Sigma-Aldrich, St. Louis, MO, USA), and 1% penicillin/streptomycin (Corning). Dimethyl sulfoxide (0.5%; Carl Roth GmbH, Karlsruhe, Germany) was added to the medium to maintain the HepaRG cells in a differentiated state. The following day, the medium was renewed with HepaRG medium containing 2% dimethyl sulfoxide. The cells were maintained in this medium for 6 days until spheroid formation.

HHSteCs were expanded in stellate cell medium (ScienCell). The cells were used between passages 3 and 8. Pre-culture was commenced at least 2 days before spheroid formation.

Bronchial MucilAir cultures were received in 24-well plates, treated in accordance with the manufacturer’s protocol, and maintained in MucilAir maintenance medium (Epithelix) for 5–7 days before transfer to the Chip3plus. The medium was changed every 2–3 days, with visual inspection at each feeding to confirm tissue integrity and the presence of beating cilia.

### Liver spheroid production

Human liver spheroids were formed by combining differentiated HepaRG cells and HHSteCs in 384-well spheroid microplates (Corning) in standard HepaRG culture medium, as described by Bauer *et al*.^12^, with minor modifications. Briefly, 50 μL of medium containing 24,000 hepatocytes and 1,000 HHSteCs were pipetted into each well of an ultra-low-attachment spheroid microplate (Corning). The plate was centrifuged for 2 min at 300 × *g* and incubated at 37 °C in 5% CO_2_. Liver spheroids without HHSteCs were thawed and formed directly after thawing in HepaRG culture medium. Compact spheroids formed within 3 days. Forty spheroids were collected in a 24-well ultra-low-attachment plate (Corning) in standard HepaRG culture medium. The plate was placed on a 3D rotator (PS-M3D; Grant Instruments, Royston, UK) for 1 day before the spheroids were used for the experiments.

### Co-culture of a bronchial MucilAir culture and liver spheroids in the Chip3plus

The Chip3plus cultures were all cultivated in MucilAir maintenance medium. Forty liver spheroids were loaded into the 96-well culture compartment and cultured on glass or in a polyethylene glycol-based SpheroidBrick inlay purchased from Cellbricks. One bronchial MucilAir culture was transferred into the middle 24-well culture compartment. The third culture compartment of each circuit was used as a medium reservoir. An additional Chip3plus was loaded with one bronchial MucilAir culture only. The Chip3plus was filled with a total volume of 4 mL medium: 400 µL of medium was added below the bronchial MucilAir culture, 700 µL of medium was added to the liver culture compartment, and the culture compartment used as a medium reservoir was filled with 2.9 mL of medium. The lung culture compartment was then sealed with a special screw (Fig. 1A) containing a gas-permeable cellulose fibre based membrane (non-woven white Rayon, Corning). Half of the medium was replaced every 2–3 days. Cultivation ended after 14 days, and the organ equivalents were collected from the culture compartments.

### On-line oxygen measurement with PreSens technology

D2 sensors were placed at the bottom of the chip during chip production directly after the bonding procedure to measure partial pO_2_ in the medium. D7 sensors were placed onto the inward-facing side of the lid for measurements in the air above the bronchial MucilAir culture. Gas-permeable membranes were used with D7 or D5 sensors. The pO_2_ at the measurement locations was determined from the other side of the glass slide, lid, or gas-permeable membrane by using a fluorescence spectrometer (PreSens Precision Sensing GmbH) connected to fibre optics and analysed by using the PreSens Measurement Studio 2 software. Two-point calibration was performed for each measurement condition, and linear regression was applied. Measurements of pO_2_ were conducted for up to 14 days at various locations inside each Chip3plus (including a blank chip without any tissues). The gas-permeable lid above the bronchial MucilAir cultures was replaced by a standard lid for some experiments.

### AFB1 treatment

Chip cultures were set up and maintained with MucilAir medium for 2 days to enable the tissues to adapt to the dynamic culturing. The medium was replaced entirely with MucilAir medium containing 5 µm AFB_1_ (VWR, Radnor, PA, USA) dissolved in 0.1% dimethyl sulfoxide in all three culture compartments for AFB_1_ exposure. The medium was replaced with MucilAir medium containing 0.1% dimethyl sulfoxide for the vehicle control. The tissue cultures were harvested for further analysis after 24 h of AFB_1_ treatment.

### Analysis of medium samples

Medium samples were collected during the medium exchange every 24–72 h for albumin and LDH analysis. A commercially available ELISA kit was used to measure albumin concentrations (E80–129; Bethyl Laboratories, Montgomery, TX, USA). Absorption was determined by using the FLUOstar Omega microplate reader (BMG Labtech, Ortenberg, Germany) at 450 nm. LDH release into the medium was analysed by using the Indiko Plus analyser (Thermo Fisher Scientific, Waltham, MA, USA). Positive controls (maximum LDH release values) were generated by addition of 1% (v/v) Triton X-100 (VWR).

### Measurement of transepithelial electrical resistance

Transepithelial electrical resistance in the bronchial MucilAir cultures was measured by using an STX-2 electrode connected to an EVOM2 epithelial volt/ohm meter (World Precision Instruments, Sarasota, FL, USA) in accordance with the manufacturer’s instructions. The value was multiplied by the surface of the inserts (0.33 cm^2^) to obtain the resistance value in the total area (ohm × cm^2^).

### Measurement of ciliary beating frequency

Ciliary beating frequency was measured in bronchial MucilAir™ cultures on days 0 and 7 and at the end of the experiment. The cultures were placed on a heating plate pre-warmed to 37°C on an inverted phase-contrast microscope (Axio Ver. A1, Carl Zeiss, Oberkochen, Germany) equipped with a 10 × objective and connected to a high-speed digital video camera (acA1300, Basler AG, Ahrensburg, Germany). Ciliary beating frequency was recorded at 120 frames per second in short movies composed of 512 frames and analysed by using the SAVA analysis software (Ammons Engineering, Clio, MI, USA).

### Immunohistochemistry

Liver spheroids from each culture were embedded in Tissue-Tek O.C.T. compound (Sakura Finetek, Torrance, CA, USA) and subsequently snap-frozen for immunohistochemical analysis. Representative central cryostat sections of the liver spheroids were stained with a combined TUNEL/Ki67 immunofluorescence stain to detect proliferating and apoptotic cells. The 8-µm sections were stained by using the Apo-Direct apoptosis detection kit (Thermo Fisher Scientific) in accordance with the manufacturer’s instructions. The slides were then blocked with 10% (v/v) goat serum in phosphate-buffered saline for 20 min, treated with primary mouse anti-human Ki67 antibody (Thermo Fisher Scientific, Clone 20Raj1) overnight, washed twice, and developed by goat anti-mouse CF594 (Biotium, Fremont, CA, USA) for 45 min. DAPI (Roche Diagnostics, Basel, Switzerland) was added for nucleus staining. Images were obtained by using a Keyence fluorescence microscope (Osaka, Japan).

### ATP-based cell viability assay

The CellTiter-Glo 3D cell viability assay (Promega, Madison, WI, USA) was used to measure the total ATP content of the tissues. The bronchial MucilAir cultures were transferred to an empty 24-well plate, and 150 µL of CellTiter-Glo reagent was added on the apical side of the cultures. Single liver spheroids were collected in a 96-well clear-bottom white polystyrene microplate in 50 µL of medium, and 50 µL of CellTiter-Glo reagent was added. The plates were shaken vigorously for 5 min to induce cell lysis and then incubated for an additional 25 min at room temperature. Control wells containing culture medium without cells were prepared to obtain a value for background luminescence. Aliquots (50 µL) of each lysate were collected in a 96-well clear-bottom white polystyrene microplate for the bronchial MucilAir samples. Luminescence was measured by using the FLUOstar Omega microplate reader (BMG Labtech).

### Statistical analysis

Statistical significance was tested by using the Brown–Forsythe and Welch ANOVA tests with the Dunnett’s T3 post-hoc test (ATP values) or mixed-effects analysis with Tukey’s post-hoc test in Prism v. 8.0 (GraphPad, La Jolla, CA, USA). *p*  <  0.05 was considered statistically significant.
